# Specialism

**Published:** 1895-05

**Authors:** Nathan E. Wood

**Affiliations:** Boston


### SPECIALISM.[1]

BY REV. NATHAN E. WOOD, D.D., BOSTON.

Mr. President and Gentlemen,—I once had a deacon, a good man, who not a little amused his pastor by explaining in a conference meeting how greatly he enjoyed the pastor's preaching. He sat in his pew Sunday after Sunday, he said, and the pastor's sermons made the cold shivers run up and down his back. You will see at once that there is wide room for conjecture as to just what was his meaning. "Cold shivers up and down the back" would not at first thought seem to be an altogether pleasant Sunday recreation, yet the deacon seemed to enjoy it. His remark had such openness, and offered such scope for the imagination, that it was

[1] Read before the American Academy of Dental Science, November 14, 1894.

really delicious, and is a cherished recollection to that pastor even to this day.

I am proud to confess to you that dentists have given me a similar sensation more than once. Indeed, there are few persons whom I have known who have so great ability as dentists in making the shivers run up and down one's back; and they always have such an easy way of paralyzing one's vocabulary on those occasions that one can neither "talk back" nor describe one's feelings. It will be an easy sum in arithmetic to work,—viz., if one dentist can so affect me, what must be my feeling to be face to face with forty?

The times in which we live and are actors are peculiar, and in that respect are like all other times in which men have lived. A generation of animal life in the air, in the sea, or on the land may come and go and be differentiated in no way from what precedes or what follows. The characteristics, so far as science can discover, may be undeviatingly alike. But no generation of men has been characteristically like any other. Each generation leaves its own diverse record of character, deeds, and results, and each age differs from every other in the long procession of human history. Our age characteristics are clearly enough defined. They need sober study and understanding by every man who desires to live efficiently. One ought to know the general drift of things around him, and somewhat also of the minor currents and eddies. Doubtless some men will live efficiently without having any very clear knowledge of their own times. But I fancy that even such men distinguish, by means of an indefinite mental sensitiveness, something of what is passing about them. They guide themselves instinctively by this undefined, unclassified, and I might almost say clandestine information which is thus filtered in upon them by a kindly providence. Nature always seems especially forbearing and tender towards the improvident; nevertheless, clearsightedness and provident effort are more certain to furnish a coat to one's back as well as guiding to a true life. This century, which is now almost closed, will be labelled among all the centuries as the wealth-getter. In this particular it stands pre-eminent and unrivalled. Its energies have been turned first of all to the exploration and the mapping out of the hitherto unknown areas of the earth's surface. In this direction there is very little more possible of accomplishment. A few more ice-fields, icebergs, and barren coasts remain yet to be compassed. There are a few more polar bears whose acquaintance has not yet been made, and a few more Esquimaux to be brought into the camp of those who are curious about such matters. But practically, the earth's surface is now known. The century has bent itself also with almost superhuman energy to the mastering of material resources. Almost all the development and discoveries of the modern sciences have been made to contribute towards the increase of wealth. The problems of wealth have absorbed the thought of the age. The world has never before known such an era of invention; but invention has been transmuted always into terms of material gain.

The relations of peoples and nations to each other are finally adjusted upon terms of commercial advantage. The cabinets of the world study the tables of interest, rates of discount, and the profits of investments, and then decide upon what is best to be done or not to be done. Statesmanship stands behind a pair of gold scales, and a pennyweight decides the fate of nations. The steamship and the railway-train are the foci upon which the ellipse of the century is circumscribed. All life seems to be drawn to the scale of good bargains and rapid accumulations of property. Even the problems of society and the social order are studied almost wholly with a view either to profit and loss or to the amelioration of physical conditions. Materialism is lord of the hour. The enormous increase of wealth has opened the problem of its equitable distribution. Socialistic theories innumerable are in the air with downy wings, like thistle seeds, ready to alight and grow in any open piece of soil. These two problems have overshadowed all others in our age,—viz., how to increase wealth, and how to maintain an equal distribution. The conspicuous name of the one is commerce, and of the other, is Socialism; but both of them have to do almost exclusively with the material side of life.

I do not forget the mighty intellectual achievements of our century, nor the repeated conquests at home and abroad of the Christian religion. I do not forget the extraordinary enfranchisement of some of the down-trodden classes of society. Nevertheless, it seems to me that the words which most completely summarize, and therefore will label the century in history, are "wealth-getting."

A curious illustration of this overmastering passion for materialism is shown in the history of the doctrine of modern evolution. It began its work with physical organisms. Its investigations were in the unfoldings of physical life. Its steps were from protozoa to the body of man. It has eagerly thrust its researches into almost every phase of material organisms. It has been immensely active in study of the earth's crust and history, and of organic material forms. But it has only just fairly awaked to the study of the higher life in man. It has seemed almost as if evolution recognized no higher life than the concourse of physical atoms called the human body. It seemed bent on dethroning both heart and intellect in man, or rather, perhaps, of wholly ignoring their existence. Indeed, whichever way one looks to watch the intenser activities of our time, one sees that the main convergence of them is upon material things.

Political economy is one of our newer and rapidly-developing sciences. It is peculiarly popular. It would seem that it ought to be broad enough to cover the whole body politic, the complete "politeia," but one finds that it concerns itself, even on the sociological side, chiefly with wealth. The laws, the protection, the increase and the distribution of wealth are the chapters of its study. The State would appear to exist as an organized system only for the protection of property. Legislation is chiefly concerned about property. Under our beneficent system, a man steals a handkerchief from your overcoat pocket, or a loaf of bread from a baker's cart, and he is straightway thrust into prison. He debauches body and soul by whiskey sold over a counter, or by foul literature in clandestine print or upon the "bill boards;" he appeals to the most debasing passions, and cultivates the ruin of that which is noblest in us, and the State rises up with all its legal and police majesty to protect him. We make him an alderman, or justice of the peace, or Congressman. The goddess of liberty comes down from the dome of the State House and holds her ægis before him, so that virtue, morality, and religion may be unable to smite and manacle him. Faneuil Halls ring with speeches about personal liberty and a bill of rights. In a word, touch property, and a prison yawns to receive you. Lay hands on the noblest thing which is in man and drag it down to destruction, and your reward is the applause and the votes of your fellow-citizens. If a fire starts in a warehouse in Boston, the whole fire department will turn out and work unweariedly to put out the blaze. The city is absorbed with interest in checking the conflagration. Saloons, gambling dens, bawdy houses, presses reeking with impure literature, haunts of crime, shameless indecency upon bill-boards, traffic in the bodies and souls of the young, poison the atmosphere so that virtue and character wither and die. The foul contagion strikes members of households here and there over the whole city, and yet the city sleeps quietly and in peace.

Is it not a fair inference to say that the age regards wealth as a more valuable product than life? This overwhelming prepossession in favor of property has developed another strange condition, which, perhaps, I can bring before you by illustration better than by abstract statement. The factory has grown to its present great proportions around the idea of the division of labor. In a wagon-factory you may see one man who does nothing for ten long hours every day but take spokes from a pile and hold them under a trip-hammer, which at a single stroke drives them into the hub. Another man guides the machinery chisel which makes mortices in the felloes; and another, a painter, does nothing except stripe the side boards of lumber-wagons. So through all the endless subdivisions of labor you will see men engaged in the minutest kinds of tasks. If factories are to go much farther along this line, microscopes will be needed by every artisan so that he may be able to find his own special work. The tendency of modern wealth-getting has been so rapid towards the minutest subdivision of toil that we have scarcely as yet had time to realize the effect on society at large. Labor becomes more skilled when directed within narrow limits. Articles of manufacture can be produced more cheaply and the profit of sale be made larger by this method. More perfect work is done, and our material comforts are largely increased. There can be no question but that this tendency has added greatly to the increment of wealth. Combinations of capital and subdivisions of labor are the two hands which have builded the temple of modern commercial prosperity. The economist points triumphantly to this result, and all his theories seem justified. And he is justified, if wealth-getting is all that there is in life worth living for. His conclusions are true, if the chief end of man is the hoarding of material gains. But the preacher may be pardoned, if he insistently inquires what will be the result of all this upon a man's higher life? What must be the effect upon the man himself, of these narrowed tasks upon which he is compelled to spend his time and strength? What mental habit will be begotten? What range of outlook will become fixed? Will he become near-sighted or far-sighted in regard to the great facts and truths of the higher life? Must the necessities of wealth-getting be so constraining of his attention that he will think of nothing but the work of his hands? Specialism in manual labor is good, doubtless, in so far as increase of wealth is concerned. But the artisan's mental horizon will become adjusted finally to the very small area of his own special task. Heaven becomes a mill where the machinery goes without a hitch, and hell a mill where the machinery is all awry. Thought is limited in scope to a wheel-spoke, or the polishing of a pin-head, or the filing of an amalgam. Life is bounded by the six walls of a shop or a store, and conscience becomes only a response automatically to the approval or disapproval of an overseer. The highest good to be reached is to become the most skilful possible machine for the production of the most perfect possible material product.

The two powerful tendencies of modern times are clearly against the development, the enrichment, and the ennoblement of what are to me the great elements in human life. Political economy, on the one hand, urges subdivision of labor as the true road to increase of wealth, and specialism in toil as a supreme law. This tends to make of men merely skilful machines. It treats them as material beings. Socialism, on the other hand, preaches the solidarity of the race in such a theory as destroys individualism. The individual is nothing. The social order is everything. The levelling of individuals is to proceed until uniformity of human beings shall take the place of diversity, and the dead level of a society robbed of its loftiest elements is reached. Between these millstones of wealth-getting and socialism men are to be crushed, if it is possible to crush them. They will destroy what is nobly characteristic and best in the individual human units which compose society. Both of them look at life as if it were only physical, and as if a material well-being wholly measured what are really measureless heights, depths, and areas of a man's life.

Gentlemen, a man's life ought to be larger than any work which he does. Work and life ought not to be commensurate terms on any true theory of life. My protest against specialism in labor is that it seeks to reduce work and life to the same terms, levels, and measurements. It is idle to complain of the meagreness and narrowness of the lives of workingmen, so long as society crowds them down into the narrow compass of their specialty and bids them live there. Health of the larger sort does not flourish under ground. Is it a compliment or not when you meet any man who has a specialized task and instinctively begin to converse with him upon topics which belong to his own work alone? Do you not in effect imply that the man knows nothing except in his own line of work? His life is shut up within his shop. I will talk to him of what he understands. His knowledge and measurements of life are shop-marked, and therefore I will confine myself to ideas which are shop-marked. You talk down to his comprehension. You pass over the counter of exchange of ideas only such coin of conversation as has his shop-stamp upon it, and has been specially minted for his narrow and poverty-stricken use. The same grave charge lies against professional work in almost all its departments. One dentist no longer attempts to cover the whole range of dental science. One man extracts teeth, another makes them; one man fills cavities, another prepares plates. Indeed, the subdivisions of labor have already grown very numerous. Doubtless dentistry profits by this method. Better work is done. Greater profits are gained. Scientific treatment is assured. The physical comfort of mankind is increased. Probably the increment of wealth perceptibly enlarges. But the interrogation point still calls for an answer to the question, What is the effect on the dentist himself of this continued narrowing of his calling? Is it to be followed by a narrowing range of mental and spiritual vision? Is the horizon of a doctor of dental surgery to be made coterminous with a dental cavity? Is life to have only the boundaries of the alveolar process, or to be merely a series of alveolations? These seem very simple questions, but they need very sober answers. Specialism in medicine has proceeded so far as to raise the question what we who are practised upon are to do. We are sick. We send for a physician. He is a specialist in gout. He discovers no gout about us, and therefore can do nothing for us. We send in haste for another physician. He is familiar with colic only. He finds no trace of colic in us, and is powerless to help us. Another physician comes and examines us for symptoms of jaundice, but finds the skin clear and liver active. Another comes and pokes our jaws to discover signs of mumps. He knows only mumps. At last one comes who recognizes that we have a fever. Ah, what endless possibilities in medical science does a fever open! But who can tell us what produced the fever? Heaven help us, else we may perish before the right specialist is hit upon to give us relief! Fortunate, indeed, shall we be if we can diagnose our own ailments so as to call the required specialist in time! Where is specialism to end? Is the whole mental attitude and poise of a professional man to become adjusted to his own special calling? The highest truths and the noblest interests of mankind have never yet been measured by the tape-measure of a profession or weighed in the scales of a specialist. The scalpel has not yet uncovered a thought, nor dissected a soul. The whole trend of specialism for which our century has become so justly famous is towards a narrowing measurement of life, and towards a microscopic study of human relations and destiny. The microscope has been and is a valuable servant. It has opened a marvellous realm of knowledge, and has become the beneficent auxiliary of science. It has become the close and powerful ally of all those professions which minister to the physical life of man. But the microscope will never enable a man to see the stars, and you have left off from life its dome and its climax if you do not see the stars. Professional work is of necessity largely microscopic work, and the mental chapters in the autobiography of a professional man are too often only the consecutive chapters in a work on microscopy. It is our modern method which is largely responsible for this unnatural rivalry between a man's professional work and a man's greater life. They ought to be in loving wedlock. Each ought to minister to the enrichment and ennoblement of the other. But alas! too frequently they are declared enemies, and the profession vanquishes the life and is all unconscious of what a gruesome victory it has won. A profession is, after all, only the coat which we are compelled to wear for the sake of temporary comfort and conventional decency. It will become threadbare and be flung away after a while. Is not the great Teacher's query always pertinent, "Is not the life more than meat, and the body than raiment?" What is the chief end of man? The modern catechism answers very promptly, "To gain a livelihood and hold on to it as long as possible." I take the old catechism by preference. I like its answer better,—"To glorify God and to enjoy Him forever." But successful specialism is fascinating, and withal, it must be confessed, useful. Moreover, it is an exceedingly jealous master, and insists that a man shall give to it all that he has. The tremendous competitions of modern life emphasize this demand. The beguilements both of fame and of property-getting are always about us. It is not strange that most men should finally succumb to so great allurements. The idols of wealth are niched in every street and in every market-place. We are always getting upon our knees, with hats off, before this shrine to worship, and oftener than not our knees are in the mud. Yet, after all, the most of wealth is only mud, coined and put in circulation. The mental habits of specialism are in utmost danger not only of a contractile narrowness, but also of a gross and absorbing materialism. They will transmute nobler things into their own forms. Emerson says "that Lord Coke, 'the great English jurist,' valued Chaucer highly because the Canon Teman's Tale illustrated the statute fifth, Henry IV., chapter fourth, against alchemy, and that the physician Sanctorius spent his life in a pair of scales, weighing his food." Now, all this is good professionalism, but bad life.

The higher education is tending to fall into the same drift. Courses of study are made elective, and young men make their choices along the line of a future calling. The Humanities, which broaden and fructify the mental life; Ethics, which would make the moral life virile and the conscience sagacious; Religion, which would open the windows of the soul Godward and manward; all these are passed by, and what are called practical studies (heaven save the mark!) are pursued with a perverted avidity. They are the bread-and-butter studies. The majority of men would greatly prefer to starve their minds rather than their bodies. Give us, they cry, a good steaming dinner in eight courses every day in the year, and plenty of other meals to match it. But "not a wave of trouble rolls across their peaceful breast" if they haven't had a new idea, or read a new book, or had a solitary stir of intellectual and spiritual life towards the noblest things in a twelvemonth. An expanding paunch and a contracting brain are the monstrous twins to which modern materialism has given birth. You will rightly guess that my protest is not against a thorough knowledge of one's specialty. Mastery of details as to chemical constituents, climatic conditions, suitable fertilizers, judicious crops, and thorough subsoiling of one's professional acre, every man ought to have; but let him not suppose that his acre is the earth. Life was meant to be drawn to the scale of the universe and not to the scale of an acre, even though that acre will produce for him cabbage and bread. I believe with Lowell when he says, "I had rather the college should turn out one of Aristotle's four-square men capable of holding his own in whatever field he may be cast than a score of lopsided ones developed abnormally in one direction." The danger from professional specialism is that just such "lopsided men developed abnormally" will result. A great dentist is admirable, but if he is produced at the expense of a great life, he is pitiful. I have only time to call your attention to this serious subject. Modern specialism necessitates most intense rivalries and conflicts in a man's own life. Unhappily, to most of us there is no conflict, because we quickly succumb to our profession, and its lordship over our lives becomes undisputed. But if a man will live a great, noble, growing, true life, he will find himself in the fiercest sort of a conflict. He must with hourly insistence cultivate the mental and spiritual habits of the larger view of life, of the wider horizon of thought, and of the eternal heights and depths of a soul. The encroachments of specialism upon a man's temper and range of thinking are so dangerous, the contractile power is so great, the temptations are so alluring and destroying, that every man should be open-eyed to see and avoid the danger. The habit of wide and varied reading in diverse sections of general literature; the curiosity of the scientific mind carried into many fields of research; the devout spirit bending itself with profound thoughtfulness over the facts and problems of religion; the set purpose always standing out in the clear of our vision to live a life that shall be wide and deep and high; and the plain acknowledgment without reservations that life is not to be ephemeral, but is to be interwoven with the life of God; all these in us will hold life in its higher levels, and faithful to its true goals. Goethe makes the world-spirit say to Faust, "Du gleichst dem Geist den du begreifst" ("Thou art like the spirit which thou dost understand").

Gentlemen, and I do not forget that the best dentists are always *gentle*-men, may your success in your chosen calling be great, but may that success be only a segment of the larger areas of your lives!

